# Exploring Mycosporine-Like Amino Acids (MAAs) as Safe and Natural Protective Agents against UV-Induced Skin Damage

**DOI:** 10.3390/antiox10050683

**Published:** 2021-04-27

**Authors:** Anjali Singh, Mária Čížková, Kateřina Bišová, Milada Vítová

**Affiliations:** Laboratory of Cell Cycles of Algae, Centre Algatech, Institute of Microbiology of the Czech Academy of Sciences, Novohradská 237, 379 81 Třeboň, Czech Republic; singh@alga.cz (A.S.); cizkova@alga.cz (M.Č.); bisova@alga.cz (K.B.)

**Keywords:** ultraviolet-B radiation, skin diseases, mycosporine-like amino acids (MAAs), sunscreen, MAAs-Np conjugates

## Abstract

Prolonged exposure to harmful ultraviolet radiation (UVR) can induce many chronic or acute skin disorders in humans. To protect themselves, many people have started to apply cosmetic products containing UV-screening chemicals alone or together with physical sunblocks, mainly based on titanium–dioxide (TiO_2_) or zinc-oxide (ZnO_2_). However, it has now been shown that the use of chemical and physical sunblocks is not safe for long-term application, so searches for the novel, natural UV-screening compounds derived from plants or bacteria are gaining attention. Certain photosynthetic organisms such as algae and cyanobacteria have evolved to cope with exposure to UVR by producing mycosporine-like amino acids (MAAs). These are promising substitutes for chemical sunscreens containing commercially available sunblock filters. The use of biopolymers such as chitosan for joining MAAs together or with MAA-Np (nanoparticles) conjugates will provide stability to MAAs similar to the mixing of chemical and physical sunscreens. This review critically describes UV-induced skin damage, problems associated with the use of chemical and physical sunscreens, cyanobacteria as a source of MAAs, the abundance of MAAs and their biotechnological applications. We also narrate the effectiveness and application of MAAs and MAA conjugates on skin cell lines.

## 1. Introduction

Over recent decades, due to the depletion of the stratospheric ozone layer and by increasing anthropogenic pollution, the levels of UV radiation are increasing gradually over the Earth’s surface. Depending on the wavelength, physical properties and biological activities, UV radiation is arbitrarily divided into three main categories: UV-C radiation (100–280 nm), UV-B (280–320 nm) and UV-A (320–400 nm). UV-A radiation is further divided into two sub-categories, longwave UV-A1 (340–400 nm) and shortwave UV-A2 (320–340 nm). Prior to their reduction by the Earth’s atmosphere, UV-A, UV-B, and UV-C represent 6.3%, 1.5% and 0.5–1.0% of total solar irradiance, corresponding to an intensity of 85.7, 21.7 and 6.4 Wm^−2^, respectively [1,2]. Among these, highly energetic UV-C radiation and almost 90–95% of UV-B radiation does not reach the ground due to absorption by the stratospheric ozone layer and losses through atmospheric scattering. Solar UV-B radiation reaching the Earth surface represents about 1–10% of total radiation, whereas almost all UV-A radiation (~95%) reaches the ground [1,3,4]. A declining ozone column leads to an increase in the intensity of UV-B reaching the Earth’s surface, and the wavelength composition is also proportionally shifted towards shorter wavelengths, which are more dangerous [1,5]. Such increases in UV-B radiation are most pronounced in the Antarctic, but similar trends have also been recorded at other latitudes [5,6].

It is well documented that excessive exposure to UV radiation has notable negative impacts on the growth, metabolism and reproduction of all living organisms, ranging from animals and plants to microorganisms [7]. Besides exerting several adverse effects on terrestrial vegetation and plankton, UVR can also penetrate deeply into human skin and induce a sequence of skin damaging cascades. Elevated exposure of UVR can be acute or chronic and can cause several skin diseases such as erythema, edema, hyperpigmentation, immune suppression, photo-aging and skin cancer [8,9]. The effect of UV radiation on the skin epidermis depends on the range and intensity of UVR. However, continuous exposure to UV radiation can lead to structural and functional changes in the skin epidermis. It can cause degradation of collagen fibers, lesions and pigmentation, and further promote photo-aging and cancer [10,11]. Several studies have shown that expression of photo-aging-linked genes such as integrin and procollagen are also associated with UV-induced skin damage [12]. Over recent decades, to mitigate the damaging effects of UVR, the use of chemical and inorganic sunscreens has accelerated. However, long-term exposure to these sunscreens has also been shown to cause several skin-associated disorders, leading to skin aging, skin cancer or other neurological disorders [13]. There is, therefore, an urgent need to identify natural and effective sunscreen materials such as MAAs that have the potential to protect against UV-induced damage and can enhance the efficacy of natural sunscreens by forming conjugates with nanoparticles or biopolymers. In response to this need, this review critically summarizes UV-induced skin damage, the use of and problems associated with chemical, physical or inorganic sunscreens, the abundance and properties of MAAs, and the efficacy of MAAs to protect against UV-induced skin damage. This study may provide new insights into our understanding of how MAAs may be a better and safer alternative than the use of cytotoxic sunblocks.

## 2. Damaging Effects of UVR on Skin

**Figure 1:**
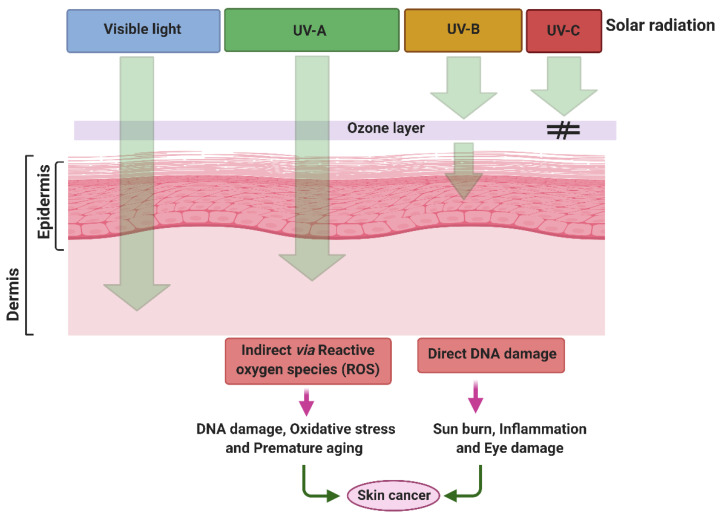
Penetration of UV radiation into the skin layer and their adverse effects. Created with BioRender (https://biorender.com/ accessed on 15 April 2021).

**Figure 2:**
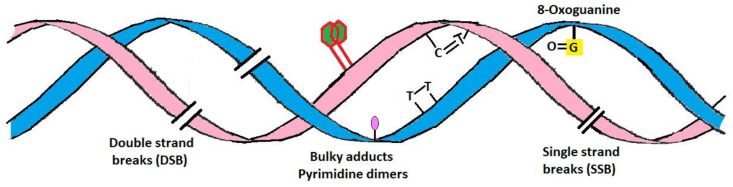
UV-induced direct and indirect DNA damage. Formation of thymine cyclobutane dimers (CPD) and (6-4) pyrimidine-pyrimidone (6-4 PP) photoproduct.

Among the different types of UV radiation, UV-A is much more predominant globally in sunlight than UV-B but is less erythemogenic and is assumed to be proportionally less responsible for the development of carcinomas. Nevertheless, UV-A irradiation can penetrate the dermis of the skin by up to 1 mm and leads to the generation of oxygen free radicals; these cause modifications to DNA bases, specifically 8-oxo-7,8 dihydroguanine, which is directly linked with the development of skin cancer [14,15]. UV-B, however, is a minimal but highly energetic component of the solar spectrum, is mutagenic, carcinogenic and is commonly known as burning rays. UV-B can penetrate the epidermal layer of skin up to 160–180 nm and is considered to be the most active constituent of solar radiation. As compared to UV-A radiation, UV-B is 1000 times more active in causing sunburn [16,17]. UV-B can induce direct and indirect adverse effects on biological molecules such as DNA and proteins by affecting the formation of pyrimidine photoproducts, isomerization of trans- to cis-urocanic acid, stimulation of DNA synthesis, the formation of free radicals, photo-aging and photo-carcinogenesis (Figure 1). DNA lesions are also significantly affected by the wavelength of incidental UV irradiation, primarily associated with the topographical location on Earth. Among several biological targets, DNA is considered one of the predominant target sites for UV-B radiation [18]. DNA lesions induced by UV radiation can occur via direct absorption of UV by the native DNA molecule or indirectly through oxidative stress. UV-B radiation can induce a variety of DNA lesions comprising dimeric photoproducts such as *cis-syn* cyclobutane pyrimidine dimers (CPDs) and pyrimidine (6-4) pyrimidone photoproducts (6-4 PPs) and their Dewar isomers, as well as dsDNA breaks (Figure 2) [19]. UV-B also plays a key role in inducing skin cancer (squamous and basal cell carcinomas) and immunosuppression. The formation of CPDs and 6-4 PP induced by UV-B irradiation mediates the modification of DNA bases, which leads to dimerization reactions, mainly among adjacent pyrimidines [20,21,22]. Increased formation of thymine dimers led to higher mutation rates resulting in the development of skin cancers [20].

Mutagenesis and the death of skin cells symbolize the critical cellular effects induced by UV irradiation on human skin [15,23]. Similarly, DNA damage can be indirectly induced by sunlight after photon absorption via other chromophores, generating reactive oxygen species that can oxidize nucleotide bases. The resulting inhibition of DNA damage repair and defective repair of DNA photoproducts are linked with aging and skin cancer, respectively. The protein linked with DNA damage repair has a faster repair response in young rather than in older adult fibroblasts cells [24,25,26]. Ultimately, aging is the most significant risk factor in the induction of skin cancer. Over the last 2–3 decades, there has been a significant increase in the incidence of skin cancers, and indeed most of the cases were associated with UV irradiation. Broadly, skin cancers are divided into melanomas and nonmelanomas, the latter including basal and squamous cell carcinomas (BCC and SCC, respectively). BCC and SCCs are commonly found in UVR exposed regions and especially on facial and neck areas [13,27], where exposure is linked significantly to the intensity of UVR and skin pigmentation of the population. A study showed that both BCC and SCC have a higher incidence on legs of women compared to men due to the greater exposure of women legs to UVR [27]. In addition, the risk of acquiring a BCC is four-fold higher than an SCC, although a SCC has a 10-fold higher risk of metastasis and mortality [28]. In recent decades, a few reports show that about 65–90% of all melanomas were due to UVR exposure [29]. Although the increasing occurrence of skin cancer is affected by several factors, UVR is regarded as a potent carcinogen and critical factor. It is one of the greatest threats to human health [13,27].

### Application of Sunscreen Against UV-Induced Skin Damage

Every organism has several defense mechanisms to mitigate UV irradiation, such as the synthesis of UV-absorbing compounds [30]. Melanocytes, specialized epidermal cells, produce melanin, a skin pigment that protects skin against detrimental UV rays. However, melanin protection is never enough because it absorbs only 50–75% of total UVR, especially in the summer season when high energy UVR can cause acute sunburn [30]. Nowadays, apart from necessity, application of sunscreen has become a trendy strategy to protect the human skin from the harmful effects of UV irradiation. It comprises one or numerous artificial UV filters that block incident UV light penetration of the skin epidermis. Sunscreens are broadly classified into physical and chemical agents. For example, physical sunscreens have the power to reflect and scatter UV-B, UV-A and visible radiation. However, chemical sunscreens generate heat during their response in dissipating UV radiation, which has adverse effects on human skin. Besides, most chemicals only block a narrow section of the UV spectrum. Therefore, in general, chemical sunscreens contain several chemical constituents, each one blocking a different part of the UV spectrum, although most are active in the UV-B region [31]. The most commonly used chemical filters comprise a variety of active ingredients: butyl methoxydibenzoylmethane (avobenzone), benzophenone-3 (oxybenzone), terephthalylidene dicamphor sulfonic acid or ecamsule (mexoryl SX), ethylhexyl or octyl salicylate (octisalate), p-octyl-methoxycinnamate (cinnamates), trolamine salicylate, 3,3,5-trimethylcyclohexanol (homosalate), 2-ethylhexyl 2-cyano-3,3-diphenylprop-2-enoate (octocrylene), bis-ethylhexyloxyphenol methoxyphenyl triazine or bemotrizinol (tinosorb S), bis-benzotriazolyl tetramethylbutylphenol or bisoctrizole (tinosorb M), para-aminobenzoic acid (PABA) and its esters, and ethylhexyl methoxy-cinnamate (octinoxate) [32,33,34]. Among these ingredients, the uses and cytotoxic effects of a few chemicals are briefly described below, along with physical sunblocks i.e., TiO_2_ and ZnO_2_.

Avobenzone: It is a dibenzoylmethane derivative and one of the most commonly used broad-spectrum sunscreen chemicals that can completely block UV-A radiation; however, it is considered to be an unstable chemical because it can lose about 50% of its efficacy when it exposed to light. That is why it is mostly paired with other chemicals or with TiO_2_ and ZnO_2_. In the US, the FDA has allowed up to 3% avobenzone in any sunscreen formulations, but regular use of avobenzone is linked with skin cancer [35]. Over the last few years, there has been an increasing trend to mix synthetic UV filter molecules like avobenzone (Parsol^®^1789) with other chemicals. Nevertheless, it has only limited use due to its adverse effects. In several other countries, the use of combinations of avobenzone and physical sunscreens are prohibited. Another reason behind the limited use of avobenzone is its instability in the absence of physical sunscreens [36].

Oxybenzone: Another regularly found chemical in most broad-spectrum sunscreens can block both UV-A and UV-B irradiation. It belongs to the class of aromatic ketones and is also known as benzophenone-3; up to 6% is permitted in sunscreen formulations in the US. Several reports showed that oxybenzone can be absorbed by the skin, leading to photoallergies, endocrine disruption, organ system toxicity, neurotoxicity, and contact allergies [37,38,39].

Mexoryl SX: Mexoryl SX is a benzylidene camphor derivative and is frequently used in combination with avobenzone, which increases the stability of both compounds. It can filter UV light, especially UV-A1 radiation associated with skin aging, and is commonly present in sunscreens and other body lotions [40].

Octisalate: This is an oil-soluble sunscreen that gives protection against only UV-B radiation but gets degraded when exposed to sunlight, so it is usually used in combination with other UV filters. It is an ester made by the condensation of salicylic acid with 2-ethylhexanol and is commonly known as ethylhexyl salicylate or octyl salicylate.

Homosalate: This organic sunscreen belongs to a class of salicylates that absorb only UV-B rays and prevent direct skin damage by sunlight. Like other chemicals, it also has harmful effects. It acts as a potential endocrine disruptor and is linked with hormone disruption [41]. Along with direct health concern, homosalate exposure can enhance pesticide absorption in the body [42,43]. It is not readily degraded and persistent in our environment [41,44].

Octocrylene: Octocrylene is an ester that belongs to the cinnamates family. It has the ability to absorb both UV-A and highly energetic components of UV-B radiation and is present in sunscreens and other body care cosmetic products up to a maximum concentration of 10%. It also has allergic and toxic effects, especially on aquatic organisms [45,46,47].

Tinosorb S and M: This is a diarylmethane and is commonly used in chemical sunscreens that can give protection against both long and short wavelength UV-A and UV-B radiation. It also provides stability to other UV filters, and up to 10% is allowed in sunscreen formulations, making it suitable for sunscreens [35,48].

PABA: This is one of the first widely used UV-B filters, but it is no longer used extensively. Due to the rising threat of allergic dermatitis and photosensitivity, the popularity of PABA-containing sunscreens has reduced over the recent decades. Up to 5% of PABA is allowed in sunscreen formulations. However, several European countries have imposed a ban on its use because their toxic effects at this concentration have been recorded in several animals [49,50].

Octinoxate: This is an ester formed by methoxycinnamic acid and 2-ethylhexanol. It is a common and effective UV-B filter, helping to protect against both sunburn and aging in combination with avobenzone. It is known by numerous different names such as methoxy-cinnamate, OMC or ethylhexyl methoxy-cinnamate, and 7.5% is permitted in sunscreen formulations. Octinoxate is associated with neurotoxicity, reproductive toxicity, and endocrine disruption, like oxybenzone. Unexpectedly, octinoxate has also been found in urine, blood and breast milk [38,51,52,53,54,55,56].

The use of synthetic and organic sunscreen agents containing these ingredients has several known side effects such as photosensitization, photoirritation and contact dermatitis. The use of organic chemicals in sunscreens is therefore not considered safe for the long-term. TiO_2_, ZnO_2_ and CeO_2_ based sunscreens are more favored because they are considered less harmful than organic sunscreens. The most widely used physical sunblocks are TiO_2_ and ZnO_2_, which protect against a broad spectrum of UV-B and UV-A radiation. The use of TiO_2_ in sunscreens give it scattering power over a broad-spectrum [57,58].

Titanium Dioxide: This exists naturally in the Earth’s crust in the form of nanoparticles. It is a broad-spectrum UV filter that can absorb UV-B and some UV-A rays but is not effective against long wavelength UV-A1 radiation. It is one of the most widely used physical sunblocks present in sunscreens and is found in sun protection factor (SPF) makeup products, lotions, and skin lightening products. TiO_2_ is generally considered safe and effective, although several studies show that oral consumption could be carcinogenic to humans [41,59].

Zinc Oxide: Zinc oxide is also considered as a naturally present broad-spectrum UV filter. It can provide protection against both UV-A as well as UV-B radiations. Up to 25% of zinc oxide is permitted in sunscreen formulations. Research has shown that it is safe for regular use on human skin, but it has some toxic effects on aquatic organisms. If it is orally inhaled or swallowed, then it can also be harmful to humans. It is considered photostable and useful for sensitive skin. Nevertheless, studies suggest that it is not as effective as chemical sunscreens and is not effective against sunburn [41,60].

Over the last decade, the use of nanoparticle based products has become popular for users as well as for producers, leading to an increase in the use of products such as sunscreens based on nanodiamonds, nanotubes and nanoparticles of TiO_2_ (TiO_2_-Np), CeO_2_-Np or ZnO_2_-Np [61,62,63]. However, the potential effects of nanoparticles on human or environmental health have not been appropriately evaluated. Hitherto, researchers have not adequately understood the possible impacts of nanoparticles. The nanoparticles are reported to be chemically reactive and more bioavailable due to their small size, which makes it difficult to track them in the body. Microfine TiO_2_- and ZnO_2_-containing sunscreens therefore also have no long-term guarantee of safety. Ultimately, these can reach aquatic ecosystems through the producer, user or disposal, and causes harmful effects on life associated with the aquatic ecosystem. The toxic impact of TiO_2_-Np has been studied by Abe et al. [64] on *Tetraselmis* sp., a phytoplanktonic organism, and found that a dose of more than 25 mgL^−1^ induced precipitation, agglomeration, cell disruption and other harmful effects [64]. Over recent decades, it has been noticed that the long-term use of physical and chemical sunblocks has led to a dramatic increase in cases of skin cancer, demonstrating the inadequacy of natural sunscreen agents and emphasizing the need to find an alternative.

To mitigate the harmful effects of physical and chemical sunscreens, naturally-derived molecules have received considerable attention as photoprotective compounds. Photosynthetic and nitrogen-fixing cyanobacteria have one of the most effective UV protection mechanisms, i.e., synthesis of secondary metabolites such as MAAs. MAAs, are a group of naturally occurring compounds found in a wide range of prokaryotic and eukaryotic organisms [65,66]. MAAs can absorb UV radiation and act as a photo-protectant due to their structural and functional characteristics. They have the potential to scavenge reactive oxygen species (ROS). MAAs are well-known photo-protectants because of their ability to act as direct and indirect antioxidants, anti-inflammatory and osmoregulatory agents that provide exciting platforms for developing a natural sunscreen [67]. Nowadays, in addition to their effective UV-screening capacity, MAAs have attracted attention in both industrial and pharmacological fields because of their potential anti-aging effects and skin cell regenerative activity. A few recent reports have shown that MAAs protect skin against UV-induced skin damage by reactivating UV-suppressed expression of genes [68,69,70].

## 3. Mycosporine-Like Amino Acids

Mycosporine-like amino acids, commonly known as “MAAs” represent a diverse family of more than 40, small <400 Da, water-soluble, colorless UV-absorbing compounds that protect against highly energetic UV photons. They have a unique absorption spectrum with a single, narrow band with an absorption maximum between 309 and 362 nm. Structurally, MAAs are divided into two groups; (i) the mycosporines, which have a single modified amino acid residue connected to a cyclohexenone core, and (ii) MAAs, have two such amino acids substituents [71]. MAAs have a 5-hydroxy-5-hydroxymethyl-cyclohex-1, 2-ene ring structure, and a methoxy-substituent in C2 position. In the MAA structure, the C3 position is always substituted with an amino group, whereas the C1 position can be substituted with either an oxo- or an imino-moiety. In some instances, the term ‘mycosporine’ refers to those with a ketone group at the C1 position, known as oxo-mycosporines or mono-substituted mycosporines.

In contrast, the term MAA is typically used for those with an imino-group like imino-mycosporines and bi-substituted mycosporines [65,66,72,73]. The structure of mycosporine, specifically the ring substitution by an oxo- or an imino-moiety, determines the absorption maximum of a particular compound. A study by Whitehead and Vernet, ref. [74] demonstrated that depending upon the oxo- and imino-moiety, the oxo- or mono-substituted mycosporines, namely mycosporine-glycine (mycosporine-Gly), mycosporine-taurine, mycosporine-alanine, mycosporine-serine, and mycosporine-serinol, showed an absorption maximum at 310 nm in the UV-B range whereas imino-mycosporine absorbs in the UV-A region (Table 1). MAAs can dissipate energy as heat without generating ROS [75]. Mostly MAAs are stable over a wide range of temperatures (up to 60 °C) and pH (up to pH-11), but these are not the same for every MAA. The presence of flavins and air can cause light-mediated photolysis of mycosporine-glutamine into amino cyclohexenone, and 2-hydroxy glutaric acid [76].

### 3.1. Progression of MAAs as UV-Screening Compounds and Their Distribution

The presence of MAAs has been reported in a wide range of living organisms, including invertebrates and vertebrates such as cyanobacteria, red and green algae, dinoflagellates, fungi, lichens, corals, sponges, sea urchins, scallop and fish [77,78,79,80,81,82,83,84,85,86,87,88,89,90,91]. Nevertheless, the evolution of UV-screening compounds is a subject that has not been extensively studied. Mulkidjanian and Junge, [92] hypothesized that aromatic-groups bearing reaction center compounds were the earliest UV-screens that subsequently started to perform a light-harvesting role in photosynthesis. The mechanism behind the origin of UV-screening compounds is still undiscovered, although it is assumed that initially, many photoprotective compounds evolved for other biological functions but later developed UV-screening functions under selection pressure [93]. Besides the photoprotective role, MAAs also functioned as osmotic regulators, especially where a hypersaline environment surrounded the cyanobacterial cell. Probably, such roles may have given rise to the first UV screening MAAs [93,94,95]. To provide the necessary osmotic balance, most cyanobacteria accumulate MAAs as “osmotic solutes” or “compatible solutes” in the cell’s intracellular space, which builds osmotic pressure within the cell. MAAs naturally accumulate as solutes in the cytoplasm, but their derivatives, covalently bound to oligosaccharides, can be excreted into the enveloping ‘sheath’ (glycocalyx), as in *Nostoc commune* [96]. Generally, MAA production is stimulated by UV-B radiation. Nevertheless, it is suggested that as oxygen levels increased in cells, UV-A screening, mainly with di-substituted MAAs, became important because most of the effects of UV-A are mediated through oxygen-free radicals [97]. This change in the absorption spectrum from UV-B to UV-A can be achieved by replacing the ketone group with a nitrogen atom in UV-B absorbing compounds. This has a more significant mesomeric effect on the benzene ring, and absorbance is shifted into the UV-A region. A mutation in a UV-B screening compound’s proposed biosynthetic pathway may have also caused a shift towards UV-A absorption [98]. In cyanobacteria, the MAA content can be correlated with a moderate physiological amelioration of photo-damage, which persists even under conditions of physiological inactivity, as expected for a sunscreen [99]. Exposure to intense solar radiation induced or enhanced the accumulation of MAAs in most of the MAA-synthesizing organisms, although the exact action spectrum for their responses varies [100].

### 3.2. Diversity of MAAs

The common mono- and di-substituted MAAs identified from cyanobacteria are listed in Table 1 with their molecular weights, molar extinction coefficients and absorption maxima [65]. Because cyanobacteria arose in the Precambrian era and have been exposed to high evolutionary pressure, they had the potential to produce MAAs, which, in contrast to other organisms, protect them against diverse environmental conditions. The occurrence of MAAs has been recorded in all diverse habitats, especially in those that are exposed to extensive solar radiation, desiccation, as well as high temperature and other stresses. Similarly, cyanobacteria exposed to intense solar radiation such as in paddy fields, on the bark of trees, roof-tops etc., have been isolated and have been shown to contain mycosporine-Gly and shinorine, with a role in photoprotection [78,101]. Some common types of MAAs such as asterina-330, shinorine, and mycosporine-Gly have been reported to play a significant photoprotective role in freshwater cyanobacteria of the high-mountain lakes situated in Austria’s Central Alps [102]. Similarly, the bloom-forming cyanobacterium, *Microcystis aeruginosa,* synthesizes shinorine and porphyra-334, which absorb UV-B radiation and thus allows the cyanobacterium to develop and maintain surface blooms, even in the presence of high solar irradiance, including ultraviolet radiation [77]. Oren [94] first reported the presence of high levels of MAAs in the halophilic cyanobacterial community [94]. They found a very high intracellular concentration of about 98 mM MAAs in a community of unicellular cyanobacteria inhabiting a gypsum crust developing on the bottom of a hypersaline saltern pond in Eilat, Israel. Rastogi et al. [103] showed the presence of different MAAs in a cyanobacterial mat collected from the old temple located in the Phra Nakhon district, Bangkok, Thailand. The cyanobacterial mat was found to contain shinorine, porphyra-334, mycosporine-Gly, and palythiol, along with two unknown MAAs having absorption maxima at 320 and 329 nm. The mat included the presence of *Synechocystis* sp., *Scytonema* sp., *Nostoc* sp., *Gloeocapsa* sp., and *Gloeocapsopsis* sp. [103]. Singh et al. [78] have described the presence of mycosporine-Gly, shinorine and a few unidentified MAAs in different cyanobacterial crust samples collected from the rice-field, roof-top and bark of trees growing on the campus of Banaras Hindu University, Varanasi, India [78].

The accumulation of MAAs in vertebrates such as fish is the best example of symbiosis. Fish lack the appropriate biosynthetic pathways and therefore accumulate MAAs via their algal diet or by bacterial or algal symbionts. Besides the dietary source, de novo synthesis of the MAA precursor compound, gadusol, was observed in coral and fish [90,104,105]. The *Pocillopora capitate* coral has a wide range of MAAs including mono- and di-substituted MAAs such as mycosporine-Gly, porphyra-334, shinorine, mycosporine-methylamine-serine, palythine-serine, mycosporine-methylamine-threonine, palythine, and palythine-threonine [106].

### 3.3. Biosynthesis of MAA

**Figure 3:**
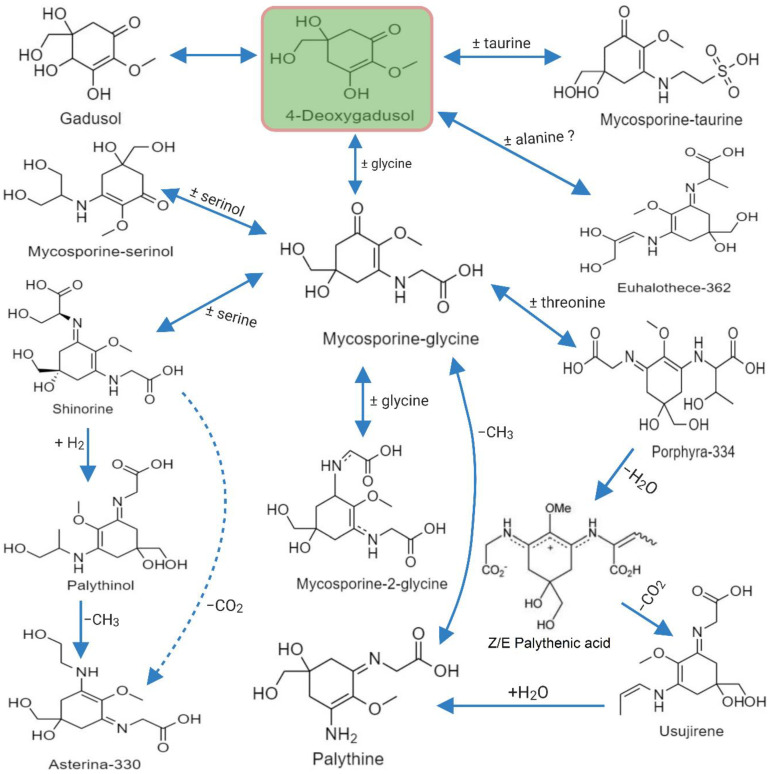
MAAs precursor and formation of a few common mono- and di-substituted MAAs.

Earlier studies suggested that the biosynthesis of MAAs arose in fungi and cyanobacteria from the first part of the shikimate pathway, probably directly from 3-dehydroquinate synthase (DHQ synthase encoded by aroB) via 4-deoxygadusol [107,108]. Studies on *Chlorogloeopsis* sp. revealed that condensation of the cyclohexenone ring, in terms of amino acids, could lead to the formation of new substituents for MAAs. Mycosporine-Gly is the first oxo-mycosporine converted into imino-mycosporines by chemical or biochemical modifications, more precisely via amino acid substitution [73,75,83]. This is depicted in Figure 3 showing how mycosporine-Gly was the first MAA synthesized, and this acted as a precursor for other mono- and bi-substituted imino-mycosporines, such as mycosporine-glycine-valine, shinorine and porphyra-334 [107].

A study of comparative genomics of four cyanobacteria has simplified the identification of the MAA biosynthetic gene locus [109]. Genome mining revealed that *Anabaena variabilis* PCC 7937 and *Anabaena* sp. PCC 7120 had two sets of the 3-dehydroquinate synthase (DHQS) gene, whereas *Synechocystis* sp. PCC 6803 and *Synechococcus* sp. PCC 6301 had a single set of this gene in their genome. In *Anabaena variabilis* PCC 7937, the cyclohexenone core was possibly formed by dehydroquinate (DHQ) synthase homologues (locus: Ava_3858), which are flanked by a putative O-methyltransferase (O-MT) (locus: Ava_4386) [109]. Likewise, the cyanobacterium *Anabaena variabilis* ATCC 29413 a known producer of shinorine possesses a putative gene cluster consisting of four ORFs. It is thought that the DHQS homolog Ava_3858 and O-MT Ava_3857 could assemble into 4-deoxygadusol (4-DG), which is a cyclohexenone core product and precursor of mycosporine. Other two ORFs such as the ATP-grasp homolog Ava_3856 and the NRPS-like enzyme Ava_3855, perhaps accountable for the assignment of glycine and serine to precursor molecules via imine linkages.

Another type of biosynthetic pathway was identified by studying *Nostoc punctiforme* gene products, where 4-deoxygadusol was produced in vitro when the culture was supplemented with sedoheptulose phosphate (SHP) as a substrate, but not when supplied with 3-DHQ. In *Nostoc punctiforme,* a demethyldeoxygadusol synthase (DDG synthase) was encoded by NpR5600 and Ava_3858, although demethyldeoxygadusol methyltransferase (O-MT) were encoded by Ava_3857 and NpR5599, respectively. These results show that this pathway branches out from central metabolism rather than from the early shikimate pathway. The Ava_3858/Ava_3857 couple and Ava_3856, a three-gene core, is highly conserved in cyanobacteria, as well as in fungi, and is essential for the biosynthesis of a mono-substituted MAA (mycosporine-Gly) from central metabolites (Gly and sedoheptulose-6-phosphate (SHP). Heterologously expressed Ava_3855 formed shinorine from mycosporine-Gly and serine in vitro, and the Ava_3855 enzyme is likely to activate serine carboxylate to catalyze imine formation. However, it remains to be seen how the cyanobacteria that lack Ava_3855 homologues achieve the synthesis of imino-mycosporines [71,72].

### 3.4. Methods Employed for Characterization of MAAs

Identification and characterization of MAAs have been accomplished by using several techniques that can determine their unique structure. The most commonly used methods for structural determinations of MAAs are UV-Vis spectroscopy, HPLC, ESI-MS, LC-MS, FTIR, NMR spectra such as 1D (1H and 13C) and 2D (COSY, NOSY and HMBC). A few other methodologies such as chemical assays (amino acid composition after alkaline hydrolysis, methylation and interchange H/D), melting point (m.p.) determination, elemental analysis, optical activity, and X-ray diffraction have also been used to characterize MAAs [73]. As mentioned earlier, one of the most significant features of MAAs is the occurrence of a unique, strong absorption peak in the UV region. Due to this exceptional characteristic feature, extracted MAAs are commonly characterized by diode-array detection (DAD) followed by HP-TLC plates or HPLC/UPLC columns [110]. Sometimes characterization and quantification of MAAs are performed via reverse-phase DAD columns with a TFA/ammonium mobile phase to increase the polarity separation of MAA mixtures [111]. Over recent decades, several mass spectroscopic techniques have been used to elucidate the structure of MAAs [112], especially since the analysis of fragmentation patterns has become more reliable. NMR always remains the method of choice for the prediction of MAA structures. These are well-conserved, consisting of a cyclohexenone or cyclohexenimine ring substituted with amino alcohols or amino acids [110]. The characteristic absorption peak of FTIR spectra of MAA indicates the presence of several functional groups such as hydroxyl, amino (NH_2_), carboxyl (COOH) and suggest the structure of MAAs [113]. The crystal, molecular structure and absolute configuration of palythene and palythine were unambiguously determined by X-ray analysis [114,115]. Recently, Orfanoudaki et al. [116] determined the crystal structure of a shinorine hydrate from single-crystal X-ray diffraction and its absolute configuration was established from anomalous-dispersion effects. Along with this, they have also determined the absolute configuration of 14 MAAs by combining the results of electronic circular dichroism (ECD) experiments with advanced Marfey’s method using LC-MS.

### 3.5. Mycosporine-Like Amino Acids and Their Applications

**Figure 4:**
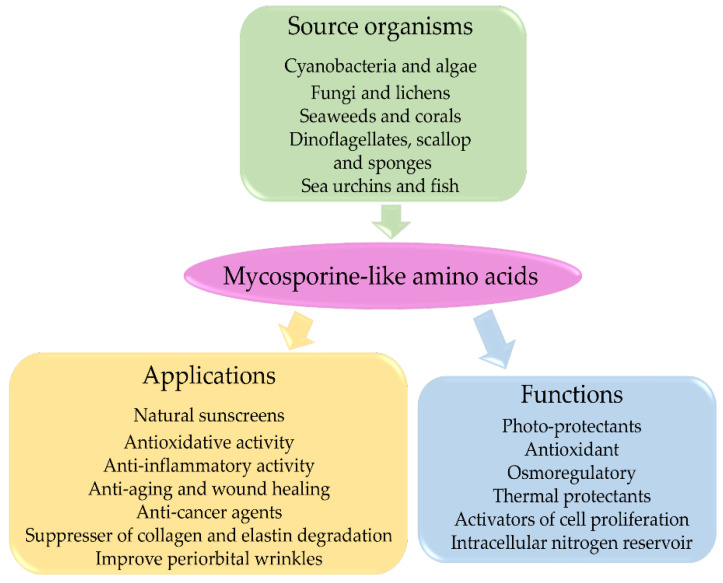
A scheme to represent MAA-producing organisms, biological functions of MAAs and their cosmetic applications.

MAAs are considered multipurpose metabolites with various functions, including antioxidant and anti-inflammatory activities, accessory pigments in photosynthesis, nitrogen storage, thermal protection, osmotic stress protection, anti-aging, anti-cancer, and wound healing. They have also been widely accepted as UV-A and UV-B photoprotection agents [93]. The evidence supporting these functions of MAAs is mostly indirect and is based on induction of MAA production following stress conditions. MAAs have well-documented applications in cosmetics, toiletries, as UV protectors, and activators of cell proliferation and as suppressors of UV-induced aging in human skin [66,67]. MAAs appear to be promising compounds in artificial sunscreens for future biotechnological research [117]. Some of their properties are briefly discussed below, and Figure 4 shows a diagrammatic representation MAA applications.

#### 3.5.1. Antioxidative Properties of MAAs

UV radiation interacts with oxygen and other organic compounds and can generate oxidative stress by producing highly reactive oxygen intermediates such as superoxide (O_2_^•−^), hydroxyl radical (^•^OH) or hydrogen peroxide (H_2_O_2_). To counteract the damaging effects of oxidative stress, cyanobacteria exhibit several defence mechanisms, such as the production of MAAs [68]. Several in vitro analyses of MAAs suggest that different abiotic stresses such as temperature, salinity, desiccation and acidity may significantly increase their antioxidative properties [81,93,118]. Oren [94] has clearly described an osmotic role of MAAs in a hypersaline environment. The antioxidative activities of mycosporine-Gly and shinorine have been very effective against ROS scavenging, although mycosporine-Gly has the highest antioxidative activity among several naturally occurring compounds. Compared with ascorbic acid, an eight-fold higher antioxidant activity has been reported at pH 8.5 for mycosporine-Gly, which was isolated from the marine lichen *Lichina pygmaea* [84,119]. The activity of porphyra-334, as an antioxidant on human skin fibroblasts, was also studied, and results showed a dose-dependent reduction in intracellular UV-A-induced ROS generation based on a modified DCF-DA fluorescence assay [119]. Recently, Kageyama and Waditee-Sirisattha [118] compiled a list of a few mono- and di-substituted MAAs as ROS scavengers, both in vitro and in vivo. Mycosporine-2-glycine has antioxidative effects in lipopolysaccharide (LPS)-stimulated RAW 264.7 macrophages by down-regulated *Sod1, Cat,* and *Nrf2* expression [120]. Ryu et al. [119] showed that treatment of porphyra-334 decreased the UV-A-induced intracellular ROS generation in human skin fibroblasts in a dose-dependent manner. Likewise, palythine isolated from the red alga *Chrondus yendoi* significantly reduced SSR-irradiated (20 J cm^−2^) production of oxidizing species in HaCaT immortal human keratinocytes [68]. Recently, RNA-sequencing analysis of human follicle dermal papilla (HFDP) cells following treatment with porphyra-334 proved the antioxidant role of porphyra-334 by upregulating metallothionein (MT)-associated gene expression [121].

#### 3.5.2. Anti-Inflammatory Properties of MAAs

Inflammation is a physiological defense mechanism to fight against molecular and cellular damage caused by oxidative stress or UV irradiation. UV mediated inflammatory responses includes synthesis of inducible NO synthase (iNOS), nitric oxide (NO), cyclooxygenase-2 (*COX-2*), prostaglandin E2 (PGE_2_), tumor necrosis factor-α (TNF-α), and other cytokines, such as interleukin-1 (IL-1) and interleukin-6 (IL-6) [118]. Expression of the *COX-2* protein, linked with PGE_2_ production, is upregulated by UV-B exposure and ROS generation in both human skin and cultured human keratinocytes [118]. Suh et al. [69] studied the anti-inflammatory response of mycosporine-Gly, porphyra-334 and shinorine against UV exposure on HaCaT cell lines. They evaluated expression levels of the *COX-2* gene (linked with tissue inflammation) under three different concentrations of MAAs (0.03, 0.15, or 0.3 mM). Results showed that mycosporine-Gly and shinorine can inhibit the expression of the *COX-2* gene, whereas porphyra did not show any significant effect [69]. Becker et al. [122] studied the immunomodulatory effects of shinorine and porphyra-334 in the human myelomonocytic cell line THP-1 and their descendent reporter line THP-1-Blue by observing activation of transcription factor NF-κB. Both cells were exposed to the MAAs in the presence or absence of lipopolysaccharide (LPS). They observed that both MAAs had immunomodulatory effects on NF-κB activity in unstimulated THP-1-Blue cells, whereas the activity of NF-κB was increased by shinorine in a more pronounced and dose-dependent manner. In contrast to this, the activity of NF-κB was reduced following porphyra-334 treatment and confirmed its anti-inflammatory potential. Likewise, Tarasuntisuk et al. [120] also reported anti-inflammatory activity in mycosporine-2-glycine lipopolysaccharide (LPS)-stimulated RAW 264.7 macrophages. Transcriptional analyses of this study showed that mycosporine-2-glycine significantly repressed the expression of *iNOS* and *COX-2*. Consequently, it also inhibited the generation of inflammatory intermediaries by suppressing the NF-κB pathway. Similarly, aqueous extracts of *Gracilariopsis longissima* and *Hydropuntia cornea* induced both TNF-α and IL-6 production in macrophages of cell line RAW264.7. These results demonstrate the anti-inflammatory properties of MAAs, particularly palythine, asterina-330, shinorine, porphyra-334, and palythinol, which are present in cell extracts of *H. cornea* and *G. longissima* [123].

#### 3.5.3. Anti-Aging and Wound Healing Properties of MAAs

Prolonged UV exposure leads to skin aging by degrading collagen fiber and reducing elastin content. Like anti-inflammatory activity, Suh et al. [69] also demonstrated MAAs efficacy against aging. They found that UV irradiation strongly suppressed expression of elastin and the procollagen c-endopeptidase enhancer (PCOLCE) gene, which binds to procollagen and enhances procollagen c-proteinase activity. However, the presence of mycosporine-Gly, porphyra-334, and shinorine elevated the UV-suppressed levels of PCOLCE and elastin in a concentration-dependent manner and also showed their wound healing properties [69]. Recently, Rui et al. [124] studied the anti-aging properties of a mixture of porphyra-334 and shinorine on ICR mice and demonstrated that MAAs have the potential to suppress collagen and elastin degradation; this could therefore be an effective treatment against skin aging. Similarly, Ryu et al. [119] showed that porphyra-334 suppressed ROS generation and the expression of matrix metalloproteinases (MMPs) that are linked with connective tissue degradation during photo-aging. At the same time, it increases the levels of procollagen, type I collagen and elastin to help maintain healthy skin cell and the healing of fibroblasts [119]. They found that porphyra-334 can suppress the expression of MMP in a dose-dependent manner. The highest concentration of porphyra-334 (40 μM) inhibited up to 56.2% MMP-1 mRNA expression in UV-A exposed human skin fibroblasts [119]. Recently, Kim et al. [121] proved the role of porphyra-334 as an anti-aging agent, including the promotion of collagen formation, improvement of periorbital wrinkles, and promotion of cell proliferation, in the human cell lines human Detroit 551 fibroblast cells, HaCaT cells, and HFDP cells derived mainly from normal human scalp hair follicles [121]. They observed that procollagen expression levels (PIP) increased in Detroit 551 cells with increasing levels of porphyra extract and porphyra-334. One ppm of porphyra extract and porphyra-334 increased the PIP content by 121% and 130%, respectively. In contrast, 10 ppm of porphyra extract and porphyra-334 increased PIP content by 147% and 154%, respectively. The in vitro efficacy of few common MAAs against UV-induced damage are listed in Table 2.

#### 3.5.4. Photo-Protective Properties of MAAs

**Figure 5:**
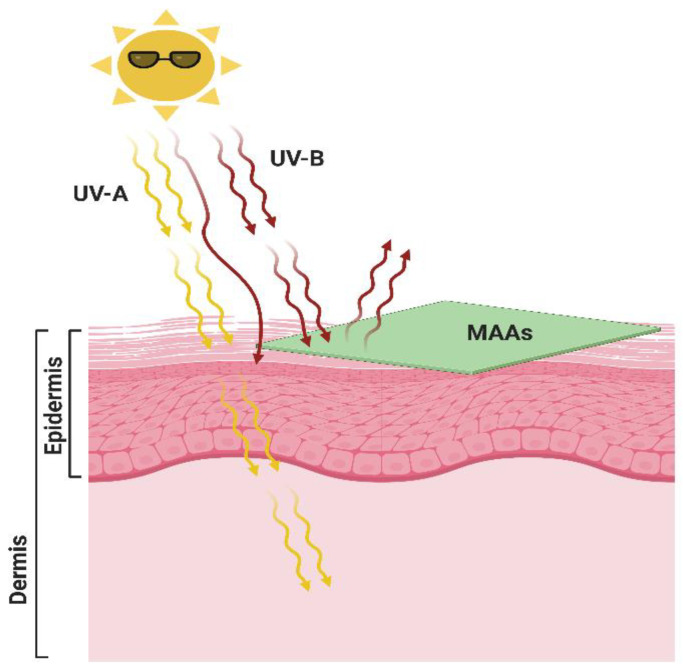
Use of natural eco-friendly mycosporine-like amino acids (MAAs) as a green sunscreen to protect skin against UV-induced skin damage. Created with BioRender (https://biorender.com/ accessed on 15 April 2021).

Over recent years, the application of MAAs in sunscreen products has attracted increasing interest. Various properties of MAAs, such as strong UV-absorption maxima (310–365 nm), high molar extinction coefficients (ε = 28,100–50,000 M^−1^cm^−1^), photostability, ability to prevent UV-induced thymine dimer formation and resistance to several abiotic stressors demonstrates that MAAs are potent photo-absorbing compounds [72,73,117]. The ability of MAAs to absorb UV radiation and dissipate energy as heat without generating reactive photoproducts makes it significant photoprotective compounds (Figure 5). Torres et al. [86] demonstrated the UV-B photoprotective activity of a novel mycosporine collemin A isolated from *Collema cristatum,* a lichenized ascomycete [86]. The photoprotective roles of porphyra-334, shinorine, and mycosporine-Gly isolated from *Patinopecten yessoensis* ovaries has been studied in human skin fibroblast cells by Oyamada et al. [80]. They found that all three MAAs can protect the cells against UV-induced cell death, but the most substantial effect was shown by mycosporine-Gly. Besides this, MAAs also promoted the proliferation of human skin fibroblast cells [80]. Lately, another interesting study conducted by de la Coba et al. [125] showed that natural sunscreen formulations combining porphyra-334 and shinorine act nearly as well as OMC synthetic UV-A filters and mycosporine-serinol as a UV-B filter. The results showed that each natural and artificial sunscreen formulation exhibited comparable SPFs when applied in identical concentrations as UV-A and UV-B filters [125].

Similarly, Moline et al. [85] showed that the photoprotective activity of yeast involved the synthesis of mycosporine-glutaminol glucoside (MGG). In this work, they analyzed the relationship between MGG production, cell survival after UV-B irradiation, formation of CPDs, photostability and singlet oxygen quenching activity of MGG [85]. Their results showed that CPD accumulation and MGG accumulation were inversely related. The conclusion of their work was that MGG plays an important role as a UV-B photoprotective metabolite in yeasts by protecting against direct DNA damage and probably against indirect damage by singlet oxygen quenching. Likewise, porphyra-334 isolated from *Porphyra yezoensis* exhibited a protective effect on human skin fibroblasts against exposure to UV-A radiation. Cell viability was increased in a dose dependent manner similar to 40 µM porphyra-334 that increased cell viability by up to 88% [119]. Recently, Suh et al. [70] used porphyra-334 to minimize the UV-induced apoptosis of HaCaT cell lines. Likewise, they also showed that UV-absorbing compounds (M-Gly, shinorine and porphyra-334) modulated gene expression associated with oxidative stress, inflammation, and skin aging caused by UV [69]. Álvarez-Gómez et al. [123] have investigated the effect of algal cell extracts of *G. longissima* and *H. cornea* on two human and one murine cell lines and found that they had no cytotoxic effects on human cell lines. Nevertheless, murine cell lines exhibited cytotoxic effects linked to immunomodulatory roles. The algal extracts included five different MAAs: palythine, asterina-330, shinorine, porphyra-334, and palythinol. They also found that the photoprotective capacity of the algal extracts in terms of SPF values showed a gradual increase with extract concentration. Both algal extracts induced the production of TNF-α and IL-6 [123].

## 4. Stability and Enhanced Effectiveness of MAA-Based Sunscreens, and MAA-Conjugates

Fernandes et al. [126] made an effort to enhance the UV protective properties of MAAs by grafting them with a chitosan (CS) matrix through amide bond formation based on carbodiimide coupling. Results showed that CS-MAA conjugates, as an extraordinarily stable combination of natural biological molecules, exhibited various benefits such as being biodegradable, biocompatible, thermoresistant, photoresistant, and with increased efficacy against both UV-A and UV-B radiation compared to individual MAAs [127]. This provides an opportunity to further engineer conjugates to generate new multifunctional materials through the modification of several remaining free amino groups on a CS matrix. It also fulfills the current requirements for cosmetic products or biopharmaceutical agents because the carbodiimide-based grafting procedure and products are already used extensively in these fields. This study provides more emphasis on the applicability of CS-MAA conjugates to a wide range of applications in fields such as cosmetics, artificial skin, wound healing, contact lenses, artificial cornea, textiles, food, drug packaging, and coatings [126]. Similarly, Singh et al. [79] have attempted to synthesize a stable ZnONPs conjugate with the UV-absorbing compound shinorine (10 mM concentration) at pH-7. They found that ZnONPs–shinorine conjugate treatment reduced in vivo ROS generation by up to 75% in *Anabaena* strain L31. Schmid et al. [128] introduced a Helioguard^®^365 commercialized natural sunscreen formulation of porphyra-334 and shinorine, extracted from the red alga *Porphyra umbilicalis.* Helioguard^®^365 has anti-aging as well as photoprotective capacity against UV-A induced skin or DNA damage. It improved cell viability in a dose-dependent manner; for example, 0.25% Helioguard^®^365 increased cell viability by up to 97.8%. In addition, 3% and 5% of Helioguard^®^365 reduced DNA damage of IMR-90 human fibroblasts following exposure to UV-A radiation. A study conducted in vivo on ten human subjects showed that Helioguard^®^365 (2% concentration) boosted the SPF value of sunscreen from 7.2 to 8.2 [129]. Likewise, twenty women volunteers applied a 5% concentration of Helioguard^®^365 twice a day. After four weeks this treatment prevented the appearance of lines and wrinkles on the skin and improved firmness and smoothness by 10% and 12%, respectively. This commercial product has broad stability such that it can be stable for 3 months at temperatures ranging from 4 to 37 °C [66,128]. In addition, Torres et al. [86] determined the SPF value of a formulation of collemin A and olive oil, in a ratio of 1:10, on the inside forearm of a volunteer at a concentration of 6 µg·cm^−2^. Fifteen minutes after application, the treated area was exposed to four MEDs (360 mJ cm^−2^) of UV-B radiation and 24 h later erythema was observed. They observed that in comparison to the positive control, i.e., octinoxate commercial sunscreen, this formulation was equally effective. However, the reliability of this study is low because only a single volunteer was involved. de la Coba et al. [125] studied the SPF value of a sunscreen formulation containing porphyra-334 (+shinorine) as a UV-A filter, and mycosporine-serinol as a UV-B filter in the ratio of 4.1:2.9%, respectively. They observed that separately these MAAs had SPF values between 4 and 6. However, in combination, the value increased to 8.37 ± 2.12, which is quite similar to the value of 9.54 ± 1.53 for the reference sunscreen formulation of avobenzone (UV-A) and octinoxate (UV-B) in a ratio of 4.5:2.6%, respectively. This study was performed in vitro [125]. Likewise, the SPF values of the algal extracts (containing palythine, asterina-330, shinorine, porphyra-334 and palythinol) of *H. cornea* and *G. longissima* showed a gradual increase with increasing concentration of extract. The highest values of SPF were recorded at 13.9 mg DW of algae per cm^−2^ which was 7.5 for *G. longissima* and 4.8 for *H. cornea* [123]. Helionori^®^ is another commercially active natural sunscreen product that includes palythine, porphyra-334 and shinorine and was extracted from the red algae *P.* *umbilicalis.* This product exhibited UV-A protective effects on human fibroblasts and keratinocytes cell lines, and, like 2% Helionori^®^, increased cell viability by up to 57 and 135% in cultures of human keratinocytes and fibroblast cell lines, respectively. This product has excellent stability against exposure to light and temperature and it can also provide a maximum level of protection to DNA by preserving membrane lipids [66,82,130].

By making comparisons over the past few decades, it is clear that the use of naturally synthesized products has significantly augmented and replaced chemical-based products. Along with other bio-based products, MAAs have also gained the attention of several researchers and industries. The application of MAA-based sunscreens may be an efficient and safer alternative for health products and cosmetics. As discussed in the previous paragraph, there are very few commercial MAA-based sunscreens on the market, so there is still a long way to go to gain acceptance of naturally derived sunscreens such as MAAs.

**Table 2:** In vitro efficacy of a few common MAAs against UV damage in skin cells.

MAAs	Skin Cell Lines	UV Radiation	Efficacy of MAAs	References
Palythine	HaCaT human keratinocytes	Solar-simulated radiation (SSR)-5–20 J cm^−2^ or UV-A radiation-20 J cm^−2^	0.3–10% *w/v* of MAAs inhibited SSR (20 J cm^−2^) induced cell death	[68]
Porphyra-334	HaCaT human keratinocytes	UV-radiation	0.1 mg mL^–1^ of porphyra-334 increased the survival rates by up to ~88%	[70]
Porphyra-334	Human skin fibroblasts (CCD-986sk)	10 J cm^−2^ of UV-A light	0–40 µM of porphyra-334 were applied, cell viability increased in a dose-dependent manner. 40 µM of porphyra-334 increased cell viability up to 88%; it also helped in wound healing	[119]
Mycosporine-Gly	HaCaT human keratinocytes	23 mW cm^−2^ between 300–400 nm. Irradiated for 15 min (275 kJ m^−2^) in UV	COX-2 expression decreased in the presence of a high concentration of mycosporine-Gly (0.3 mM)	[69]
Shinorine	HaCaT human keratinocytes	23 mW cm^−2^ between 300–400 nm. Irradiated for 15 min (275 kJ m^−2^) in UV	COX-2 expression decreased in the presence of the lowest concentrations of shinorine (0.03 mM)	[69]
Helioguard^®^365 formulation of porphyra-334 and shinorine	HaCaT human keratinocytes	UV-A (320 nm) with 10 J cm^−2^ intensity	Concentrations of 0.125% and 0.25% of Helioguard^®^365 improved cell viability in a dose-dependent manner; 0.25% Helioguard^®^365 increase cell viability by up to 97.8%.	[128]
Mycosporine-Gly, shinorine, and porphyra-334	Human normal skin fibroblast cell line TIG-114	UV irradiation with a peak at 302 nm and 0.16 mW cm^−2^ intensity.	No toxic effect of MAAs, it increased cell proliferative activity to mycosporine-Gly = shinorine > porphyra-334, and 50 mM mycosporine-Gly accelerated growth by approximately 40%.	[80]
Algal cell extracts of *H. cornea* and *G. longissima*	One murine macrophages RAW264.7, two human cell lines skin fibroblasts HGF, and HaCaT human keratinocytes	Solar simulator with a mercury-xenon lamp (51.4–63.7 W m^−2^)	The highest SPF 7.5 for *G. longissima* and 4.8 for *H. cornea*, were found at a density of 13.9 mg DW of algae per cm^−2^. Both algal extracts induced the production of TNF-α and IL-6, and they did not show cytotoxicity in human cells.	[123]
*P. yezoensis* cell extract and porphyra-334	Three different human cell lines Detroit 551, HaCaT, and HFDP cells		One ppm of porphyra-334 increased cell viability by 110.33% and 101.79%, while 200 ppm increased viability by 126.68% and 110.26% in HaCaT and HFDP, respectively.	[121]
Collemin A	HaCaT human keratinocytes	UV-B (200 mJ cm^−2^ delivered at an irradiance of 3.4 mW s^−1^)	3 mg cm^−2^ collema provide approximately 75% keratinocyte viability against UV-B radiation	[86]
Shinorine and porphyra-334	IRC mouse skin	UV-A 20.81 J cm^−2^ and UV-B 0.47 J cm^−2^	MAAs inhibited hydroxyproline reduction and protected against damage to collagen fibers in photo-aging skin. It also reduced the expression of MMP-1, MMP-3 and TNF-α.	[124]
Porphyra-334 and shinorine (11.5:1).	Mouse fibroblasts 3T3	UV-A (10 J cm^−2^).	The concentration of 0.1 to 5 µg mL^−1^ provides concentration dependent protection. Highest protection recorded at 5 µg mL^−1^ MAAs.	[131]

## 5. Conclusions

A rapid increase in skin damage in humans due to UV radiation has been reported over the past few decades, which has led to the use of many chemical/physical UV filters in order to protect skin against damage. However, a wide range of chemicals that are used to treat skin damage also have harmful effects on human health, the environment and damage to aquatic life, eventually disturbing the whole ecosystem through their bioaccumulation. In this review, we considered the use of MAAs as a natural sunscreen against skin damage, and which can be used as an adequate substitute for damaging and harmful chemicals. MAAs are known to be a functionally very diverse group of natural compounds that effectively absorb UV rays. Apart from functioning as a photo protectant, MAAs also act as anti-photoaging compounds, cell proliferation activators, anti-inflammatory or anti-cancer agents, and skin cell renewal stimulators. MAAs are now attracting commercial attention since they can provide a wide range of protection against UV rays. They conjugate with biopolymers or nanoparticles, eventually increasing their stability and effectiveness. Despite having extensive literature on the extraction and characterization of MAAs from their sources, the critical mechanisms involved in their protection against UVR has yet to be clearly addressed and is a topic for further research. Ultimately, they may become commercially available as a personalized natural sunscreen.

## Figures and Tables

**Table 1:** Mono- and di-substituted MAAs with their molecular weights (MW), absorption maxima (**λ_max_**), molar extinction coefficients (**ε**) and structures.

MAAs	λ_max_ (nm)	MW	Molar Extinction Coefficient (ε = M^−1^·cm^−1^)	Structure
Mycosporine-glycine	310	243	28,800	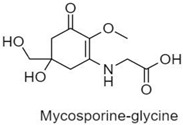
Mycosporine-taurine	309	295	28,100	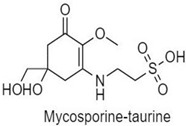
Mycosporine-alanine	310	259	640	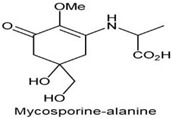
Mycosporine-serine	310	275	ND	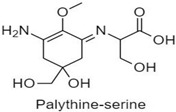
Mycosporine-serinol	310	261	27,270	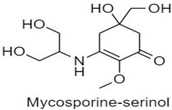
Mycosporine-2-glycine	331	302	43,800	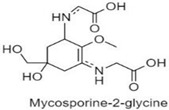
Shinorine	333–334	332–334	44,700	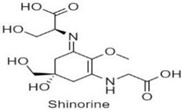
Porphyra-334	334	346	42,300	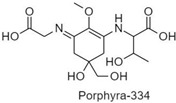
Palythenic acid	337	328	29,200	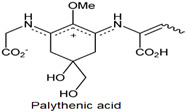
Palythene	360	284	50,000	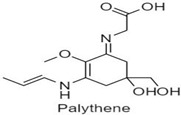
Usujirene	357	284	50,000	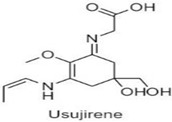
Asterina-330	330	288	43,800	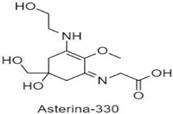
Palythinol	332	302	43,500	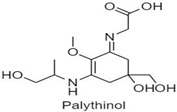
Palythine	320	244	36,200	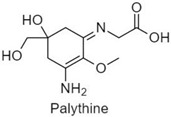
Euhalothece-362	362	330	43,800	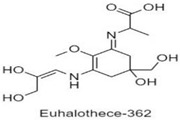

Note: The molecular weights, absorption maxima and molar extinction coefficients of MAAs has been adopted from Wada et al. [65]. ND shows that the molar extinction coefficient has not yet been determined.

## Data Availability

Not applicable.
